# Author Correction: The frequency of pathogenic variation in the *All of Us* cohort reveals ancestry-driven disparities

**DOI:** 10.1038/s42003-024-06408-x

**Published:** 2024-06-10

**Authors:** Eric Venner, Karynne Patterson, Divya Kalra, Marsha M. Wheeler, Yi-Ju Chen, Sara E. Kalla, Bo Yuan, Jason H. Karnes, Kimberly Walker, Joshua D. Smith, Sean McGee, Aparna Radhakrishnan, Andrew Haddad, Philip E. Empey, Qiaoyan Wang, Lee Lichtenstein, Diana Toledo, Gail Jarvik, Anjene Musick, Richard A. Gibbs, on behalf of the All of Us Research Program Investigators, Brian Ahmedani, Christine D. Cole Johnson, Habib Ahsan, Hoda Anton-Culver, Eric Topol, Katie Baca-Motes, Julia Moore-Vogel, Praduman Jain, Mark Begale, Neeta Jain, David Klein, Scott Sutherland, Bruce Korf, Beth Lewis, Ali G. Gharavi, George Hripcsak, Eric Boerwinkle, Scott Joseph Hebbring, Elizabeth Burnside, Dorothy Farrar-Edwards, Amy Taylor, Liliana Lombardi Desa, Steve Thibodeau, Mine Cicek, Eric Schlueter, Beverly Wilson Holmes, Martha Daviglus, Paul Harris, Consuelo Wilkins, Dan Roden, Kim Doheny, Evan Eichler, Gail Jarvik, Gretchen Funk, Anthony Philippakis, Heidi Rehm, Stacey Gabriel, Richard Gibbs, Edgar M. Gil Rico, David Glazer, Jessica Burke, Philip Greenland, Elizabeth Shenkman, William R. Hogan, Priscilla Igho-Pemu, Elizabeth W. Karlson, Jordan Smoller, Shawn N. Murphy, Margaret Elizabeth Ross, Rainu Kaushal, Eboni Winford, Vik Kheterpal, Francisco A. Moreno, Cheryl Thomas, Mitchell Lunn, Juno Obedin-Maliver, Oscar Marroquin, Shyam Visweswaran, Steven Reis, Patrick McGovern, Gregory Talavera, George T. O’Connor, Lucila Ohno-Machado, Fornessa Randal, Andreas A. Theodorou, Eric Reiman, Mercedita Roxas-Murray, Louisa Stark, Ronnie Tepp, Alicia Zhou, Scott Topper, Rhonda Trousdale, Phil Tsao, Scott T. Weiss, Jeffrey Whittle, Stephan Zuchner, Olveen Carrasquillo, Megan Lewis, Jen Uhrig, May Okihiro, Maria Argos, Brisa Aschebook-Kilfoy, Laura Bartlett, Roberta Carlin, Elizabeth Cohn, Vivian Colon-Lopez, Karl Cooper, Linda Cottler, Errol Crook, Elizabeth Culler, Charles Drum, Milton Eder, Mark Edmunds, Rachel Everhart, Adolph Falcon, Becky Fein, Zeno Frano, Michael Garrett, Sandra Halverson, Eileen Handberg, Joyce Ho, Laura Horne, Rosario Isasi, Jessica Isom, Jessica Jarmin, Megan Jula, Royan Kamyar, Frida Kleiman, Isaac Kohane, Babbette Lamarca, Brendan Lee, Niall Lennon, Dessie Levy, Todd Mahr, Emily Makahi, Vivienne Marshall, Elizabeth Mayer-Davis, Jacob McCauley, Jeffrey McKinney, David McPherson, Robert Meller, Jose Melo, David Ming-Hung Lin, Michael Minor, Evan Muse, Kapil Parakh, Cathryn Peltz-Rauchman, Linda Perez Laras, Subhara Raveendran, Gail Reilly, Jody Reilly, Nelida Rivera, Laura Rosales, Tracie Rosser, Linda Salgin, Sherilyn Sawyer, William Simonson, Amy Sitapati, Cynthia So-Armah, Gene Stegeman, Christin Suver, Michael Taitel, Kyla Taylor, Daniel Hernandez Tinoco, Jason Vassy, Jamie Walz, Preston Watkins, Blaker Wilkerson, Katrina Yamazaki, Melissa Basford, Amaryllis Silva Boschetti, Matthew Breeden, Suchitra Chandrasekaran, Cheryl Clark, Kim Enard, Yuri Fresko, Richard Grucza, Robert Kelley, Kathleen Keogh, Monica Kraft, Christopher Lough, Ted Malmstrom, Paul Nemeskal, Matt Pagel, Jeffrey Scherrer, Sanjay Skukla, Debra Smith, Bryce Turner, Miriam Vos

**Affiliations:** 1 Human Genome Sequencing Center, Baylor College of Medicine, Houston, TX USA; 2 Department of Genome Sciences, University of Washington, Seattle, WA USA; 3 University of Arizona, R Ken Coit College of Pharmacy, Department of Pharmacy Practice and Science, Tucson, AZ USA; 4 Vanderbilt University Medical Center, Department of Biomedical Informatics, Boston, MA USA; 5 Department of Pharmaceutical Sciences, University of Pittsburgh School of Pharmacy, Pittsburgh, PA USA; 6 Department of Pharmacy and Therapeutics, University of Pittsburgh School of Pharmacy, Pittsburgh, PA USA; 7 Broad Institute of MIT and Harvard, Cambridge, MA USA; 8 Department of Medicine (Medical Genetics), University of Washington School of Medicine, Seattle, WA USA; 9 Department of Genome Sciences, University of Washington School of Medicine, Seattle, WA USA; 10 NIH All of Us Research Program, National Institutes of Health Office of the Director, Bethesda, MD USA; 11 Henry Ford Health System, Detroit, MI USA; 12 University of Chicago Medical Center, Chicago, IL USA; 13 University of California, Irvine - Irvine, California, CA USA; 14 Scripps Research Translational Institute - La Jolla, California, CA USA; 15 Vibrent Health, Fairfax, VA USA; 16 University of Alabama at Birmingham, Birmingham, AL USA; 17 Columbia University - New York City, New York, NY USA; 18 University of Texas Health Science Center at Houston, Houston, TX USA; 19 Marshfield Clinic Research Institute, Marshfield, WI USA; 20 University of Wisconsin at Madison, Madison, WI USA; 21 Community Health Center, Inc., Middletown, CT USA; 22 Sun River Health - Beacon, New York, NY USA; 23 Mayo Clinic and Foundation, Rochester, MN USA; 24 Cooperative Health, South Carolina, SC USA; 25 University of Illinois at Chicago, Evanston, IL USA; 26 Vanderbilt University Medical Center, Nashville, TN USA; 27 Johns Hopkins University School of Medicine, Baltimore, MD USA; 28 University of Washington, Seattle, Washington, WA USA; 29 FiftyForward, Nashville, TN USA; 30 Broad Institute - Boston Massachusetts, Boston, USA; 31 Baylor College of Medicine, Houston, TX USA; 32 National Alliance for Hispanic Health, Washington, DC USA; 33 Verily Life Sciences - South San Francisco, California, CA USA; 34 MITRE Corporation, McLean, VA USA; 35 Northwestern University, Evanston, IL USA; 36 University of Florida, Gainesville, FL USA; 37 Morehouse School of Medicine, Atlanta, GA USA; 38 Partners Health Care - Boston, Massachusetts, MA USA; 39 Cornell University, Weill Medical College - New York City, New York, NY USA; 40 Cherokee Health Systems, Knoxville, TN USA; 41 CareEvolution, Inc, Ann Arbor, MI USA; 42 University of Arizona, Tucson, Tucson, AZ USA; 43 Delta Research and Educational Foundation, Washington, DC USA; 44 Stanford University - Stanford, California, CA USA; 45 University of Pittsburgh, Pittsburgh, PA USA; 46 Wondros - Los Angeles, California, CA USA; 47 San Ysidro Health Center - San Ysidro, California, CA USA; 48 Boston Medical Center - Boston, Massachusetts, MA USA; 49 University of California, San Diego – San Diego, California, CA USA; 50 Asian Health Coalition, Chicago, IL USA; 51 Banner Health, Phoenix, AZ USA; 52 Montage Marketing Group, Rockville, MD USA; 53 University of Utah, Salt Lake City, Utah USA; 54 HCM Strategists, Austin, TX USA; 55 Color Genomics, Inc. – Burlingame, California, CA USA; 56 NYC Health + Hospitals - New York City, New York, NY USA; 57 VA AoU Coordinating Center - Palo Alto, California, CA USA; 58 Brigham and Women’s Hospital – Boston, Massachusetts, MA USA; 59 Medical College of Wisconsin, Milwaukee, WI USA; 60 University of Miami School of Medicine, Miami, FL USA; 61 Research Triangle Institute - Research Triangle Park, Raleigh, NC USA; 62 Waianae Coast CHC, Waianae, HI USA; 63 National Library of Medicine (NLM), Bethesda, MD USA; 64 American Association of Health and Disability, Sedona, AZ USA; 65 Hunter College - New York City, New York, NY USA; 66 University of Puerto Rico Comprehensive Cancer Center, San Juan, WA USA; 67 CTSA Community Engagement Programs, Gainesville, FL USA; 68 University of South Alabama, Mobile, AL USA; 69 TPC: Blood Assurance - Signal Mountain, Tennessee, TN USA; 70 San Diego Blood Bank – San Diego, California, CA USA; 71 TPC: Denver Health - Denver, Colorado, CO USA; 72 TPC: Active Minds -, Washington, DC USA; 73 University of Mississippi Medical Center, Jackson, MS USA; 74 TPC: DLH Corp, Atlanta, GA USA; 75 Mass General Hospital - Boston, Massachusetts, MA USA; 76 Tactis -, Washington, DC USA; 77 TPC: Mary’s Center -, Washington, DC USA; 78 TPC: Owaves -, Washington, DC USA; 79 Harvard Medical School - Boston, Massachusetts, MA USA; 80 National Baptist Convention, Nashville, TN USA; 81 Gundersen Health System, La Crosse, WI USA; 82 South Texas Blood and Tissue Center, San Antonio, TX USA; 83 University of North Carolina at Chapel Hill - Chapel Hill, Chapel Hill, NC USA; 84 Sensis - Glendale, California, CA USA; 85 TPC: Bloodworks Northwest – Seattle, Washington, WA USA; 86 TPC: Fitbit - San Francisco, California, CA USA; 87 COSSMA, Aibonito, PR Puerto Rico; 88 Patients Like Me, Houston, TX USA; 89 Quest Diagnostics Incorporated – Secaucus, Secaucus, NJ USA; 90 Emory University, Atlanta, GA USA; 91 VA AoU Coordinating Center - Boston, Massachusetts, MA USA; 92 Cascade Regional Blood Services - Tacoma, Washington, WA USA; 93 ExamOne, Mission, KS USA; 94 Sage Bionetworks – Seattle, Washington, WA USA; 95 Walgreen Co., Deerfield, IL USA; 96 WebMD Health Corp – New York City, New York, NY USA; 97 Blue Cross Blue Shield, Chicago, IL USA; 98 Saint Louis University, Saint Louis, MO USA; 99 Mount Sinai Health System - New York City, New York, NY USA; 100 LifeSouth, Gainesville, FL USA; 101 SunCoast Blood Center, Bradenton, FL USA; 102 University Southern California - Los Angeles, California, CA USA

**Keywords:** Disease genetics, Molecular medicine, Genome informatics

Correction to: *Communications Biology* 10.1038/s42003-023-05708-y, published online 19 February 2024

The data availability statement was incorrectly given as “All sequencing data used in this study are available on the All of Us Researcher Workbench in the v7 release.” but should have been “All sequencing data used in this study are available on the All of Us Researcher Workbench in the v6 release”. The original Article has been corrected.

