# Supplementary material for: The frequency of pathogenic variation in the All of Us cohort reveals ancestry-driven disparities
Source: Commun Biol. 2024 Feb 19;7:174. doi: 10.1038/s42003-023-05708-y (PMC10876563; doi:10.1038/s42003-023-05708-y)
Supplement: Supplementary file 2 — Supplementary Information [file 42003_2023_5708_MOESM2_ESM.pdf]

Supplementary Tables

Supplementary Table 1: Contingency table used for chi-square test for independence. The smaller groups were aggregated into a single column for this analysis to meet the requirements of the statistical method applied. The results show that differences in the genes in which variants appear in the three largest ancestry groups (aft, amr, eur) are statistically significant ($p < 0.00001$).

	afr	amr	eur	eas, mid, oth, sas	total
HFE (hom)	5	8	238	5	256
BRCA2	44	32	122	26	224
LDLR	66	24	101	28	219
RYR1	17	16	76	11	120
BRCA1	24	9	75	11	119
MYBPC3	15	9	74	5	103
APOB	4	9	57	9	79
PMS2	22	3	46	6	77
PALB2	33	7	30	1	71
PKP2	33	6	20	5	64
MYH7	12	7	30	6	55
SCN5A	20	5	21	1	47
others	53	51	168	51	323
total	348	186	1058	165	1757

Supplementary Table 2: Genes used in the predicted loss of function (pLoF) analysis. Loss of function variants in these genes is a known disease mechanism.

Gene	Transcript	LoF Mechanism?	Dosage Score
ACTA2	NM_001613.3	NO	0

ACTC1	NM_005159.4	NO	1
APOB	NM_000384.2	NO	0
CACNA1S	NM_000069.2	NO	0
DSC2	NM_024422.3	NO	2
DSG2	NM_001943.4	NO	1
MYH11	NM_001040113.1	NO	0
MYH7	NM_000257.3	NO	0
MYL2	NM_000432.3	NO	30
MYL3	NM_000258.2	NO	0
PCSK9	NM_174936.3	NO	40
PRKAG2	NM_016203.3	NO	0
RET	NM_020975.5	NO	3
RYR1	NM_000540.2	NO	0
RYR2	NM_001035.2	NO	0
SDHAF2	NM_017841.2	NO	2
TGFBR1	NM_004612.3	NO	2
TGFBR2	NM_003242.5	NO	2
TMEM43	NM_024334.2	NO	0
TNNI3	NM_000363.4	NO	1
TNNT2	NM_001001430.1	NO	0
TPM1	NM_001018005.1	NO	0
APC	NM_000038.5	YES	3
ATP7B	NM_000053.3	YES	30
BMPR1A	NM_004329.2	YES	3

BRCA1	NM_007294.3	YES	3
BRCA2	NM_000059.3	YES	3
COL3A1	NM_000090.3	YES	3
DSP	NM_004415.2	YES	1
FBN1	NM_000138.4	YES	3
GLA	NM_000169.2	YES	3
KCNH2	NM_000238.3	YES	3
KCNQ1	NM_000218.2	YES	3
LDLR	NM_000527.4	YES	3
LMNA	NM_005572.3	YES	2
LMNA	NM_170707.2	YES	2
MEN1	NM_130799.2	YES	3
MLH1	NM_000249.3	YES	3
MSH2	NM_000251.2	YES	3
MSH6	NM_000179.2	YES	3
MUTYH	NM_001128425.1	YES	30
MYBPC3	NM_000256.3	YES	3
NF2	NM_000268.3	YES	3
OTC	NM_000531.5	YES	3
PKP2	NM_004572.3	YES	3
PMS2	NM_000535.6	YES	3
PTEN	NM_000314.6	YES	3
RB1	NM_000321.2	YES	3
SCN5A	NM_198056.2	YES	2

SDHB	NM_003000.2	YES	3
SDHC	NM_003001.3	YES	3
SDHD	NM_003002.3	YES	3
SMAD3	NM_005902.3	YES	3
SMAD4	NM_005359.5	YES	3
STK11	NM_000455.4	YES	3
TP53	NM_000546.5	YES	3
TSC1	NM_000368.4	YES	3
TSC2	NM_000548.3	YES	3
VHL	NM_000551.3	YES	3
WT1	NM_024426.5	YES	3

Supplementary Table 3: Specific predicted loss of function variant subtypes. Genes vary by the types of loss of function variants that were detected.

Gene\Consequence	frameshift variant	splice acceptor variant	splice donor variant	start lost	stop gained	stop lost
BRCA2	124	11	6	0	40	0
BRCA1	53	7	7	0	32	0
ATP7B	39	9	12	0	16	0
MSH6	42	2	5	0	19	0
PKP2	25	7	5	0	19	0
PMS2	20	7	3	0	23	0
DSP	19	3	2	0	23	0

APC	20	4	4	0	18	0
KCNQ1	18	5	7	0	15	0
MUTYH	16	6	3	0	17	0
LDLR	16	4	8	0	12	0
MYBPC3	16	6	6	0	11	0
MSH2	13	5	3	0	10	0
TSC2	13	6	2	0	8	0
SCN5A	12	2	1	0	12	0
PTEN	13	1	0	0	10	0
KCNH2	15	0	1	0	3	0
MLH1	9	3	3	0	4	0
TP53	4	6	3	0	6	0
VHL	8	4	1	0	5	0
RB1	7	4	2	0	3	0
SMAD3	11	0	0	0	3	0
LMNA	3	0	3	0	7	0
SDHD	7	1	0	0	3	0
WT1	7	0	2	0	2	0
COL3A1	1	1	2	0	6	0
GLA	8	1	0	0	1	0
SDHB	5	1	1	0	2	0
TSC1	2	3	0	0	4	0
FBN1	1	0	4	0	1	0
MEN1	3	0	1	0	2	0

SDHC	3	1	0	0	1	0
NF2	1	1	1	0	1	0
OTC	4	0	0	0	0	0
SMAD4	2	0	1	0	0	0
STK11	0	1	1	0	1	0
BMPR1A	2	0	0	0	0	0

Supplementary Table 4: ClinVar replication of findings in PKP2 and PALB2. In PKP2 and PALB2, a significant difference in the rate of pathogenic variants between the African ancestry group and the European ancestry groups was observed, using the VIP database to annotate pathogenic variants. This finding was replicated using ClinVar to annotate pathogenic variants.

Gene	Ancestry group	Ancestry group path. variants	European ancestry group path. variants	p value
PKP2	African	28 / 22,897 (0.12%)	15 / 49,668 (0.03%)	0.00002
PALB2	African	36 / 22,897 (0.16%)	30 / 49,668 (0.06%)	0.0005

Supplementary Table 5: Comparison of pathogenic rates in All of Us vs eMERGE III by disease area. Although the programs featured different gene panel designs and collection strategies, findings by disease area correlate.

	eMERGE III	All of Us
Cancer susceptibility	1.38%	0.69%
Cardiac diseases	0.87%	0.29%
Cholesterol and lipid disorders	0.50%	0.38%
hemochromatosis	0.30%	0.26%
Connective tissue / clotting disorders	0.10%	0.01%
neuromuscular diseases	0.08%	0.14%

endocrine / Immunological / Metabolic diseases	0.04%	0.01%
