# Supplementary material for: The frequency of pathogenic variation in the All of Us cohort reveals ancestry-driven disparities
Source: Commun Biol. 2024 Feb 19;7:174. doi: 10.1038/s42003-023-05708-y (PMC10876563; doi:10.1038/s42003-023-05708-y)
Supplement: Supplementary file 3 — Description of Additional Supplementary Files [file 42003_2023_5708_MOESM3_ESM.pdf]

Description of Additional Supplementary Files

File name: Supplementary Data 1

Description: Gene counts for the VIP database, which underlies the variant interpretations done in the paper.

File name: Supplementary Data 2

Description: The source data behind the graphs in the paper.
