# Supplementary material for: The frequency of pathogenic variation in the All of Us cohort reveals ancestry-driven disparities
Source: Commun Biol. 2024 Feb 19;7:174. doi: 10.1038/s42003-023-05708-y (PMC10876563; doi:10.1038/s42003-023-05708-y)
Supplement: Supplementary file 6 — Reporting Summary [file 42003_2023_5708_MOESM6_ESM.pdf]

Corresponding author(s): Eric Venner

Last updated by author(s): Aug 12, 2023

Reporting Summary

Nature Portfolio wishes to improve the reproducibility of the work that we publish. This form provides structure for consistency and transparency in reporting. For further information on Nature Portfolio policies, see our Editorial Policies and the Editorial Policy Checklist.

Statistics

For all statistical analyses, confirm that the following items are present in the figure legend, table legend, main text, or Methods section.

n/a	Confirmed	
☐	☒	The exact sample size (n) for each experimental group/condition, given as a discrete number and unit of measurement
☐	☒	A statement on whether measurements were taken from distinct samples or whether the same sample was measured repeatedly
☐	☒	The statistical test(s) used AND whether they are one- or two-sided<br>*Only common tests should be described solely by name; describe more complex techniques in the Methods section.*
☒	☐	A description of all covariates tested
☐	☒	A description of any assumptions or corrections, such as tests of normality and adjustment for multiple comparisons
☒	☐	A full description of the statistical parameters including central tendency (e.g. means) or other basic estimates (e.g. regression coefficient) AND variation (e.g. standard deviation) or associated estimates of uncertainty (e.g. confidence intervals)
☒	☐	For null hypothesis testing, the test statistic (e.g. F, t, r) with confidence intervals, effect sizes, degrees of freedom and P value noted<br>*Give P values as exact values whenever suitable.*
☒	☐	For Bayesian analysis, information on the choice of priors and Markov chain Monte Carlo settings
☒	☐	For hierarchical and complex designs, identification of the appropriate level for tests and full reporting of outcomes
☒	☐	Estimates of effect sizes (e.g. Cohen's d, Pearson's r), indicating how they were calculated

Our web collection on statistics for biologists contains articles on many of the points above.

Software and code

Policy information about availability of computer code

Data collection	Participant data were collected through the All of Us research program, with approval from the program's IRB. Data are made available through the researcher workbench. HGSCCL internal variant reviews are available through the Neptune gitlab site.
Data analysis	Data were analyzed in Hail on the research workbench. Code is available in the Demo - Assessment of pathogenic variants across the All of Us Research Program' workspace.

For manuscripts utilizing custom algorithms or software that are central to the research but not yet described in published literature, software must be made available to editors and reviewers. We strongly encourage code deposition in a community repository (e.g. GitHub). See the Nature Portfolio guidelines for submitting code & software for further information.

Data

Policy information about availability of data

All manuscripts must include a data availability statement. This statement should provide the following information, where applicable:

- Accession codes, unique identifiers, or web links for publicly available datasets
- A description of any restrictions on data availability
- For clinical datasets or third party data, please ensure that the statement adheres to our policy

All data and code reside in the All of Us Researcher Workbench in the 'Demo - Assessment of pathogenic variants across the All of Us Research Program' project on the AoU Research Workbench: https://www.researchallofus.org/. The VIP database is available on gitlab: https://gitlab.com/bcm-hgsc/neptune.

Research involving human participants, their data, or biological material

Policy information about studies with human participants or human data. See also policy information about sex, gender (identity/presentation), and sexual orientation and race, ethnicity and racism.

Reporting on sex and gender	We have used the term 'sex' to refer to the biological attribute. We do not discuss gender in this manuscript.
Reporting on race, ethnicity, or other socially relevant groupings	We have used continental ancestry groups as a means to subdivide and begin to understand how pathogenic variant rates differ by ancestry group. Understanding these differences can allow us to target future studies at specific disparities.
Population characteristics	Summaries of the cohort can be viewed in the data browser at researchallofus.org. There are currently 477,000 participants in All of Us, and the latest data release contains WGS for 245,000 participants. There are 100 partner organizations and 770 collection sites. Participants come from all 50 states. 50% are racial and ethnic minorities and 80% are underrepresented in biomedical research. 59% are women and 43% are over the age of 60.
Recruitment	Many participants are invited to enroll by one of the All of Us research program's partner health care provider organizations, which include large academic medical centers, VA medical centers, and community health centers across the United States. Participants can also enroll directly through JoinAllofUs.org, or at certain All of Us events.
Ethics oversight	Data collection for the All of Us research program is overseen by the All of Us IRB. Specific projects on the researcher workbench, including this one, are approved through the Data Resource Center.

Note that full information on the approval of the study protocol must also be provided in the manuscript.

Field-specific reporting

Please select the one below that is the best fit for your research. If you are not sure, read the appropriate sections before making your selection.

☒ Life sciences ☐ Behavioural & social sciences ☐ Ecological, evolutionary & environmental sciences

For a reference copy of the document with all sections, see nature.com/documents/nr-reporting-summary-flat.pdf

Life sciences study design

All studies must disclose on these points even when the disclosure is negative.

Sample size	The sample size was governed by the size of the data release on the All of Us researcher workbench at the time the work was carried out.
Data exclusions	No data were excluded.
Replication	We replicated some findings using an alternative source of variant pathogenicity.
Randomization	Groups were not randomized. We used genetic ancestry to divide participants into groups.
Blinding	We were not blinded to participant groupings. The outcomes of this study are objective and cannot be influenced by the participant or researcher's knowledge. Further, there was not an intervention in this study.

Reporting for specific materials, systems and methods

We require information from authors about some types of materials, experimental systems and methods used in many studies. Here, indicate whether each material, system or method listed is relevant to your study. If you are not sure if a list item applies to your research, read the appropriate section before selecting a response.

Materials & experimental systems

n/a	Involved in the study	
☒	☐	Antibodies
☒	☐	Eukaryotic cell lines
☒	☐	Palaeontology and archaeology
☒	☐	Animals and other organisms
☒	☐	Clinical data
☒	☐	Dual use research of concern
☒	☐	Plants

Methods

n/a	Involved in the study	
☒	☐	ChIP-seq
☒	☐	Flow cytometry
☒	☐	MRI-based neuroimaging
